# Mutant UBQLN2^P497H^ in motor neurons leads to ALS-like phenotypes and defective autophagy in rats

**DOI:** 10.1186/s40478-018-0627-9

**Published:** 2018-11-08

**Authors:** Tianhong Chen, Bo Huang, Xinglong Shi, Limo Gao, Cao Huang

**Affiliations:** 1 Department of Pathology, Anatomy & Cell Biology, Thomas Jefferson University, 1020 Locust Street, Philadelphia, PA 19107 USA; 2 Laboratory Animal Center, Shanxi Provincial People’s Hospital, Taiyuan, Shanxi 030012 People’s Republic of China; 3 Animal Laboratory of Nephrology, Shanxi Provincial People’s Hospital, Taiyuan, Shanxi 030012 People’s Republic of China; 4 Department of Ophthalmology, The Third Xiangya Hospital of Central South University, Changsha, Hunan 410013 People’s Republic of China

**Keywords:** Amyotrophic lateral sclerosis, ALS, UBQLN2, P62, Motor neuron degeneration, Autophagy, Rats, Protein aggregation

## Abstract

Mutations in ubiquilin2 (UBQLN2) have been linked to abnormal protein aggregation in amyotrophic lateral sclerosis (ALS). The mechanisms underlying UBQLN2-related neurodegenerative diseases remain unclear. Using a tetracycline-regulated gene expression system, the ALS-linked UBQLN2^P497H^ mutant was selectively expressed in either the spinal motor neurons or astrocytes in rats. We found that selectively expressing mutant UBQLN2^P497H^ in the spinal motor neurons caused several core features of ALS, including the progressive degeneration of motor neurons, the denervation atrophy of skeletal muscles, and the abnormal protein accumulation. Furthermore, mutant UBQLN2^P497H^ accumulation was associated with an age-dependent decrease in several core autophagy-related proteins. ALS-like phenotypes were not observed when mutant UBQLN2^P497H^ was overexpressed in the astrocytes, however, even though the expression of the mutant UBQLN2^P497H^ protein was higher in these rats. Our results suggest that selectively expressing mutant UBQLN2^P497H^ in motor neurons is sufficient to trigger the development of ALS in rats. Our results further indicate that the compromised autophagy-lysosomal pathway plays a critical role in the pathogenesis of UBQLN2-related neurodegenerative diseases.

**Electronic supplementary material:**

The online version of this article (10.1186/s40478-018-0627-9) contains supplementary material, which is available to authorized users.

## Introduction

Amyotrophic lateral sclerosis (ALS) is a fatal neurodegenerative disease characterized by the degeneration of motor neurons, progressive muscle wasting, and reduced mobility [25, 55]. Genetic studies have linked mutations in ubiquilin2 (UBQLN2) to both ALS and frontotemporal lobar degeneration (FTLD) [8, 10, 48]. How UBQLN2 mutations cause neuronal death remains to be determined.

A prominent feature of Ubqln2-linked diseases is protein aggregation [8], which is well reproduced in transgenic rats and mice overexpressing mutant Ubqln2 [10, 25, 52]. Both TDP-43 pathology and ubiquitination are common features in a variety of neurological diseases, including ALS, FTLD, and Alzheimer’s disease (AD) [31, 37]. Ubqln2-positive inclusions exist not just in patients harboring a Ubqln2 mutation but also in chromosome 9 open reading frame 72 (C9ORF72)-linked cases [5, 8]. Ubqln2 is an X-linked gene that consists of an ubiquitin-like domain (UBL) at the N-terminus and an ubiquitin-associated domain (UBA) at the C-terminus [20]. UBQLN2 shuttles between the nucleus and cytoplasm to perform functions related to protein degradation via proteasomes and autophagy [36, 41]. UBQLN2 inclusion is a well reproduced feature in in vivo models of ALS [10, 14, 25, 52]. Another ALS-linked gene, p62/SQSTM1, co-localizes with abnormal UBQLN2 inclusions [8, 10, 14, 52], suggesting a synergistic effect of UBQLN2 and p62 during neurodegenerative disease progression. Thus, accumulating evidence in both patients and rodent models suggests that Ubqln2-positive inclusions play an important role in proteinopathy in neurodegenerative disorders.

Although both mutant SOD1 and mutant TDP-43 reproduce typical ALS features in rodent models, overexpression of mutant SOD1 in spinal motor neurons does not lead to motor neuron death [29, 39], whereas selective expression of mutant TDP-43 in motor neurons causes substantial motor neuron death [16] in rats. These findings suggest different contributions of mutant SOD1 and mutant TDP43 to ALS. Previous studies have shown that mutant UBQLN2^P497H^ transgenic rat or mouse models as well as a UBQLN2^P520T^ knock-in mouse model harboring the equivalent human P506T mutation exhibited memory deficits but did not develop any phenotypes of motor neuron disease [10, 13]. It has also been shown that selective expression of either wild-type or ALS–FTLD mutant UBQLN2^P497S^ or UBQLN2^P506T^ in the motor neurons of mice leads to both memory deficits and abnormal motor phenotypes [25]. However, the influence of selectively expressing ALS-linked mutant UBQLN2 in the spinal motor neurons remains unknown.

As the most abundant glial cells in the central nervous system (CNS), astrocytes play an important role during central nervous system (CNS) development [32]. Astrocytes also serve as mediators of inflammatory responses in the CNS [45]. Recent studies suggest, however, that reactive astrocytes cause detrimental effects in neurons in several neurological disorders [2, 19, 26, 27, 35]. The contribution of astrocytes to the pathogenesis of ALS remains controversial.

Here, we show that the selective overexpression of mutant UBQLN2^P497H^ in the spinal motor neurons led to age-dependent impairment of motor functions in ChATtTA/UBQLN2^P497H^ rats, including motor neuron degeneration, skeletal muscle atrophy, progressive impairment of motor function, TDP-43 pathology, ubiquitination and glial reactions, and abnormal protein accumulation. In contrast, selective overexpression of mutant UBQLN2^P497H^ in astrocytes was not associated with motor impairment. The accumulation of p62 and ubiquitin was increased, however, in the spinal cord astrocytes of GFAPtTA/UBQLN2^P497H^ rats.

## Materials and methods

### Generation of transgenic rats

ChATtTA-9 transgenic rats [16] were crossed with TRE-UBQLN^P497H^ transgenic rats [52] to generate ChATtTA/UBQLN^P497H^ bigenic rats expressing mutant UBQLN^P497H^ in motor neurons. All rats were maintained on a Sprague-Dawley background. Similarly, GFAPtTA-line-2 transgenic rats [49] were chosen to generate GFAPtTA/UBQLN^P497H^ bigenic rats that overexpress mutant UBQLN^P497H^ in astrocytes. Multiple sets of primers were used for identifying transgenic rats: ChATtTA (5′-TGAGTTCCAGGCAAACCAAG-3′ and 5′-TCCAAGGCAGAGTTGATGAC-3′), GFAPtTA (5’-TGAGTGAGATAATGCCTGGG-3′ and 5’-ACCCTCTCTCTAGGAAGGTG-3′), and TRE-UBQLN^P497H^ (5′- AGGATCATAATCAGCCATACCAC-3′ and 5′- CTGCACCTAGTGAAACCACGA -3′). To suppress transgene expression in bigenic rats, breeding rats were fed DOX (50 μg/ml) in their drinking water during embryonic development. DOX was withdrawn from the drinking water at birth to induce the expression of mutant UBQLN^P497H^ transgene [52].

### Animal behavior tests

Mobility was tested via open-field and accelerating rotarod (Med Associates, Inc. VT, USA) tests. The total distance that a rat traveled in the open field within 10 min was recorded. The latency to fall from the accelerating rotarod (0–40 rpm) was recorded over 5 trials for 2 min per trial.

### Cresyl violet staining and cell counting

The transverse sections of the rat spinal cord (L3-L5) were dissected as previously reported [16], and stained with cresyl violet for cell counting. Using ImageJ software (NIH; Bethesda, MD, USA), both sides of every 10th section (30 μm) were counted for motor neurons larger than 300 μm^2^ (i.e., 15–20 sections per rat).

### Toluidine blue and silver staining

Rat tissues were fixed in 4% paraformaldehyde. The L3 roots of the spinal cords were dissected for toluidine blue staining. Transverse sections (5 μm) were stained in 1% toluidine blue. Using ImageJ software, the motor axons of the entire ventral roots were counted. A modified Bielschowsky’s silver stain kit (American MasterTech; Lodi, CA, USA) was used according to the manufacturer’s instructions to detect degenerating neurons. Transverse sections (10 μm) were cut by a Leica CM1950 cryostat for the staining. The degenerating neurons were stained as grey to black and normal neurons were stained as yellow to gold.

### Immunoblotting and fluorescence staining in the spinal cord

Total proteins of rat tissues were extracted with RIPA buffer and separated via SDS-PAGE for immunoblotting as described previously [16]. The resolved proteins were transferred onto nitrocellulose membranes and detected with the following targeted primary antibodies: rabbit anti-ATG7 (Cell Signaling Technology; Danvers, MA, USA), rabbit anti-LAMP2a (Abcam; Cambridge, UK), mouse anti-GFAP (Millipore; Burlington, MA, USA), mouse anti-P62 (Novus Biologicals; Littleton, CO, USA), and mouse anti-GAPDH (Abcam). The spinal cords of rats were fixed in 4% PFA and then cryopreserved in 30% sucrose for sections on the cryostat. The tissues were sectioned transversely (20 μm) and stained for the following antibodies: mouse anti-Ubqln2 (Abnova; Taipei, Taiwan), goat anti-ChAT (Millipore), rabbit anti-LC3 (Proteintech; Rosemont, IL, USA), rabbit anti-GFAP (DAKO; Lexington, MA, USA), rabbit anti-P62 (Proteintech), chicken anti-ubiquitin (Sigma; St. Louis, MO, USA) and rabbit anti-TDP-43 (Proteintech). The tissues were then incubated with the following secondary antibodies: donkey anti-rabbit IgG Alexa Fluor 488 (ThermoFisher; Waltham, MA, USA), donkey anti-mouse IgG Alexa Fluor 594 (ThermoFisher), goat anti-chicken IgY Alexa Fluor 488 (ThermoFisher), and donkey anti-goat IgG Alexa Fluor® 488 (Jackson ImmunoResearch; West Grove, PA, USA). The images were captured by a Nikon digital camera, and single layer photos were scanned with a Nikon A1R microscope confocal system (Imaging Facility of Kimmel Cancer Center at Jefferson).

### Histology and fluorescence staining for skeletal muscles

Fresh gastrocnemius muscles were snap-frozen in liquid nitrogen and cut into 10-μm thick sections on a cryostat. As previously described [16], H&E staining, nonspecific esterase, and ATPas staining were used to examine structures in the gastrocnemius muscles. Using the α-napthyl acetate protocol, nonspecific esterase activity was detected. The red-brown color revealed denervated muscle fibers, whereas yellow-to-brown color revealed normal fibers. Myosin ATPase staining pH 4.6, which revealed type 1, 2b, and 2c, and pH 10.45, which revealed type 2a and 2b, were used to detect the four types of skeletal muscle fibers. Specific mouse antibodies against myosin from the Developmental Studies Hybridoma Bank (DSHB; Iowa City, Iowa) were stained for gastrocnemius muscles along with DMD (Dystrophin, a plasma membrane marker). The images were captured by a Nikon digital camera. To reveal the integrity of the neuromuscular junctions (NMJ), 40-μm thick transverse sections of gastrocnemius muscles fixed in 4% PFA were stained for α-bungarotoxin conjugated with Alexa Fluor 594 (ThermoFisher), mouse monoclonal antibodies to neurofilament (ThermoFisher), and synaptophysin (ThermoFisher). These sections were then incubated with the secondary antibody donkey anti-mouse IgG Alexa Fluor 488 (ThermoFisher). The projected images of the NMJs were captured with a Nikon A1R microscope confocal system.

### Statistics

The numbers of motor neurons in the spinal cord or motor axons in the L3 ventral roots of the spinal cords were compared among rats using paired *t* tests. *P* < 0.05 was considered statistically significant.

## Results

### Expressing the ALS-linked UBQLN2^P497H^ mutation in motor neurons of rats

**Fig. 1:**
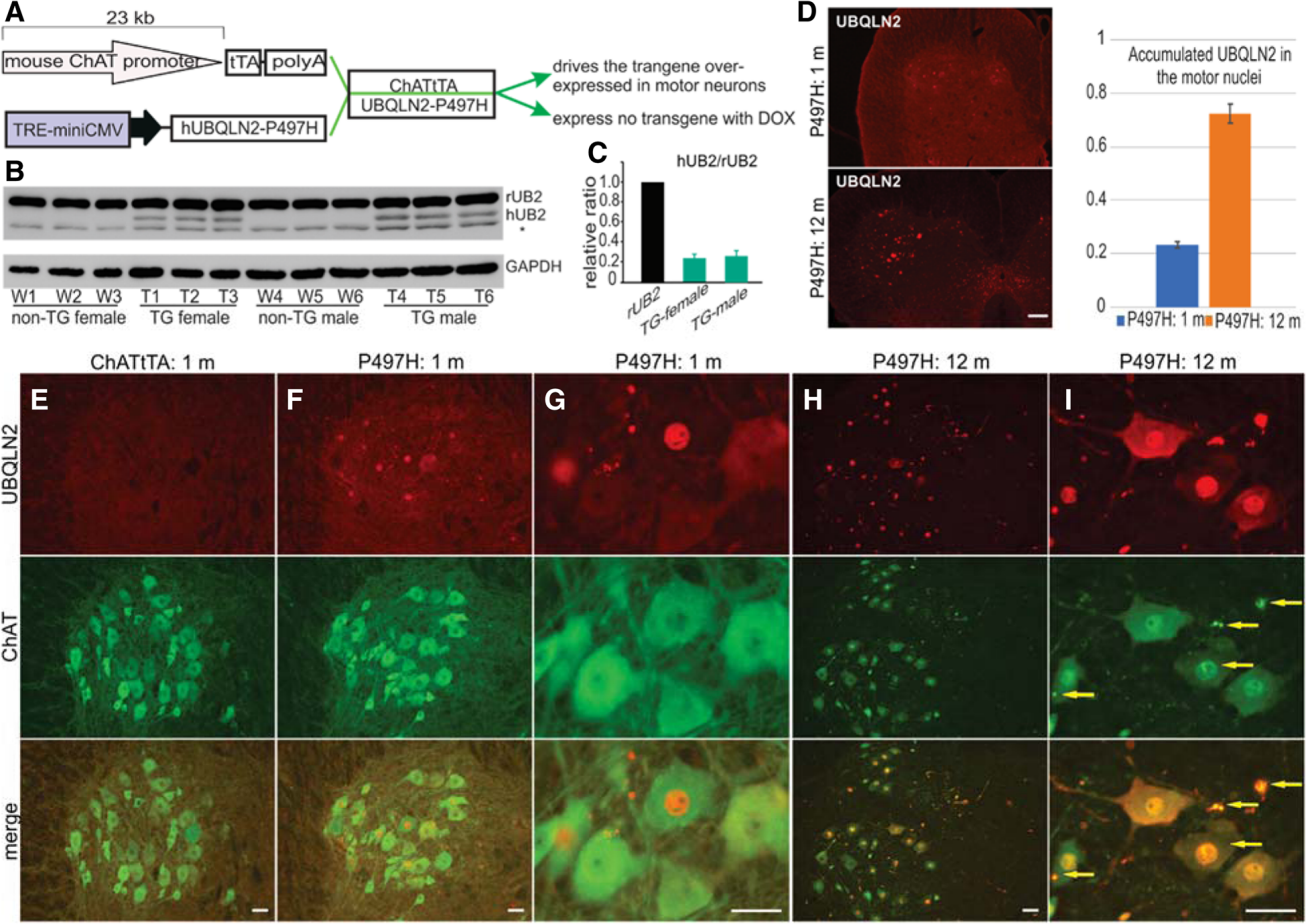
Restricted expression of the ALS-linked UBQLN2^P497H^ mutation in motor neurons of rats. **a** A graph showing the tetracycline-regulated expression system used in this study. The transgene ChATtTA binds to the TRE promoter to induce the expression of mutant human UBQLN2^P497H^. The presence of Dox suppresses the expression of the transgene. **b-c** Immunoblotting shows that human UBQLN2 was expressed in ChATtTA/UBQLN2^P497H^ (P497H) bigenic rats (TG: T1- T6) but not in control (ChATtTA) rats (non-TG: W1-W6). rUB2: endogenous UBQLN2, hUB2: human UBQLN2. The * indicates unknown bands. The data are reported as the mean ± standard deviation (*n* = 3). **d-i** Immunofluorescence staining shows that human UBQLN2 is substantially expressed in the spinal motor neurons of P497H but not ChATtTA rats. In addition, the accumulation of UBQLN2 promoted choline acetyltransferase (ChAT, a marker of motor neurons) to progressively form inclusions, which colocalized with UBQLN2 inclusions. In (**d**), a chart shows the quantification of the accumulated UBQLN2 in motor nuclei (n = 3). In (**i**), the arrows point to the ChAT inclusions. m: month. Scale bars: 200 μm (**d**); 50 μm (**e-i**)

Tetracycline-controlled transcriptional activation is a commonly used method for inducible gene expression [54]. A tetracycline-regulated expression system has proven to be a highly efficient way to regulate transgene expression in our rat models [15, 16]. As we have previously reported, expression of the ALS-linked UBQLN2 mutation (P497H substitution) in the forebrain causes progressive neuron death and learning deficits in rats [52]. We have also shown that the mouse ChAT promoter region drives the selective expression of the human TDP-43 transgene in the spinal motor neurons of rats [16], and the human GFAP promoter also works well to restrict transgene expression to the astrocytes in rats [49]. To study the effect of mutant UBQLN2^P497H^ on motor neurons, we crossed TRE-UBQLN2^P497H^ rats with the ChATtTA-9 line to produce ChATtTA/UBQLN2^P497H^ double transgenic rats that selectively express mutant UBQLN2^P497H^ in the spinal motor neurons (Fig. 1). This finding is consistent with our previous study, in which more than 70% of spinal motor neurons expressed the LacZ transgene or human TDP-43 in the ChATtTA-9 line [16].

Fifty micrograms (μg) of doxycycline (DOX) was added to the drinking water to prevent transgene expression during the prenatal stages (Fig. 1a). To induce the expression of the transgene UBQLN2^P497H^, the transgenic rats were deprived of DOX after birth. UBQLN2 is an X-linked gene [8]; therefore, we conducted Western blots independently on male and female rats, which revealed that only ChATtTA/UBQLN2^P497H^ double transgenic rats exhibited human UBQLN2 expression in the spinal cord at 30 days old. In addition, both female and male rats had similar expression levels of human UBQLN2 compared to endogenous UBQLN2, and there also was no differentiated expression of endogenous UBQLN2 between male and female rats (Fig 1b, c).

To verify whether endogenous UBQLN2 has similar expression in both male and female adult rats, different tissues were examined in non-transgenic rats at 90 days old via immunoblotting Additional file 1: Figure S1. There was no statistical difference in the expression levels of endogenous UBQLN2 between male and female adult rats at 90 days old Additional file 1: Figure S1. As described previously in ChATtTA/TDP-43 rats [16], immunofluorescence staining revealed that more than 70% of spinal motor neurons in ChATtTA/UBQLN2^P497H^ rats expressed human UBQLN2 at 12 months old compared to only 20% at 1 month old (Fig. 1d), but no accumulation of UBQLN2 in ChATtTA rats (Fig. 1e). In addition, compared to ChATtTA single transgenic rats, UBQLN2 accumulated in both the nuclei and neurites in ChATtTA/UBQLN2^P497H^ bigenic rats at 30 days old (Fig. 1f-i). In contrast, no accumulation in choline acetyltransferase (ChAT, a marker of motor neurons) was observed (Fig. 1f-g). Thereafter, ChAT was accumulated in both the nuclei and neurites of most spinal motor neurons at 12 months old, and colocalized with UBQLN2 inclusions (Fig. 1h-i). These results suggest that selectively expressing mutant UBQLN2^P497H^ in motor neurons leads to a predominance accumulation of UBQLN2 inclusions, resulting in abnormal ChAT accumulation.

### Mutant UBQLN2^P497H^ in motor neurons results in ALS-like phenotypes in rats

**Fig. 2:**
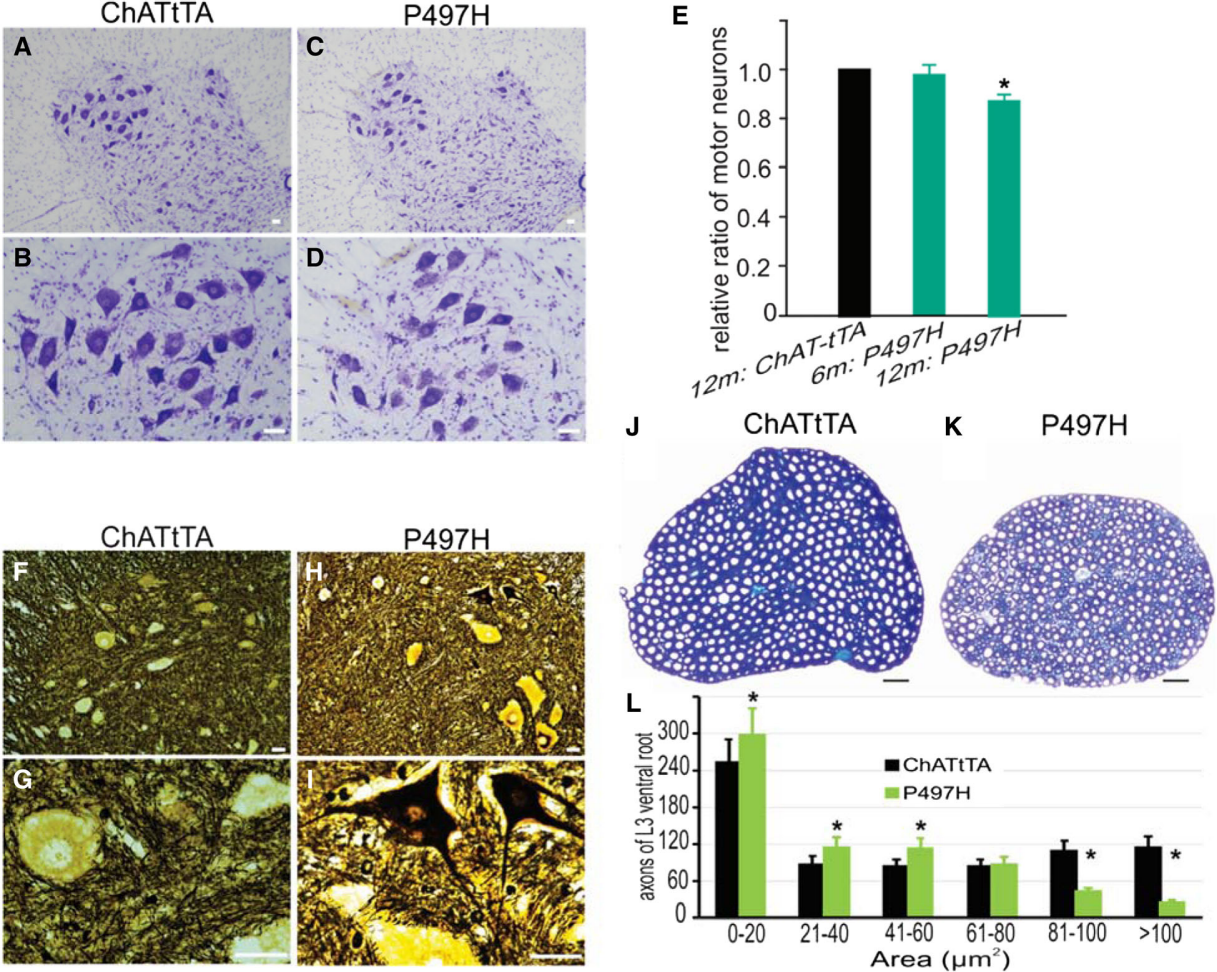
Motor neuron degeneration in ChATtTA/UBQLN2^P497H^ rats. **a-d** Representative images of cresyl violet staining revealed the motor neurons of the lumbar spinal cord. **e** A graph showing the relative ratio of the motor neurons of the L3–5 spinal cord counted using ImageJ software. This graph shows that there was a mild loss of motor neurons in ChATtTA/UBQLN2^P497H^ rats (P497H) at 12 months old. The “m” denotes month in this graph. The data are reported as the mean ± standard deviation (*n* = 4). *p < 0.05. **f-i** Bielschowsky’s silver staining showed degenerating motor neurons in the ventral horn of spinal cord in ChATtTA/UBQLN2^P497H^ but not in ChATtTA rats. **j-k** Toluidine staining revealed the L3 ventral roots of spinal cords. **l** The motor axons of L3 ventral roots were counted with ImageJ software. The data are shown as the mean ± standard deviation (n = 4 rats). Scale bars: 50 μm (**a-d**), 30 μm (**f-k**)

DOX was withdrawn at birth to induce mutant UBQLN2^P497H^ protein expression in rats. Substantial expression of mutant UBQLN2 in the spinal cord of ChATtTA/UBQLN2^P497H^ rats was observed via immunoblotting (Fig. 1). To investigate the effect of mutant UBQLN2 on motor neurons in rats, the L3-L5 regions of the spinal cord were stained with 0.5% cresyl violet (Fig. 2a-d). Neurons larger than 300 μm^2^ were counted with ImageJ software. There was a decrease in large neurons compared to ChATtTA single transgenic rats (Fig. 2e), indicating a loss of motor neurons in ChATtTA/UBQLN2^P497H^ rats. Consistent with the loss of motor neurons, there also was a substantial degeneration of motor neurons as determined via Bielschowsky’s silver staining (Fig. 2f-i). The motor axons at the L3 ventral roots were stained with 1% toluidine solution and quantified, which further revealed a dramatic loss of large axons larger than 80 μm^2^ in ChATtTA/UBQLN2^P497H^ rats at 12 months old (Fig. 2j-l).

**Fig. 3:**
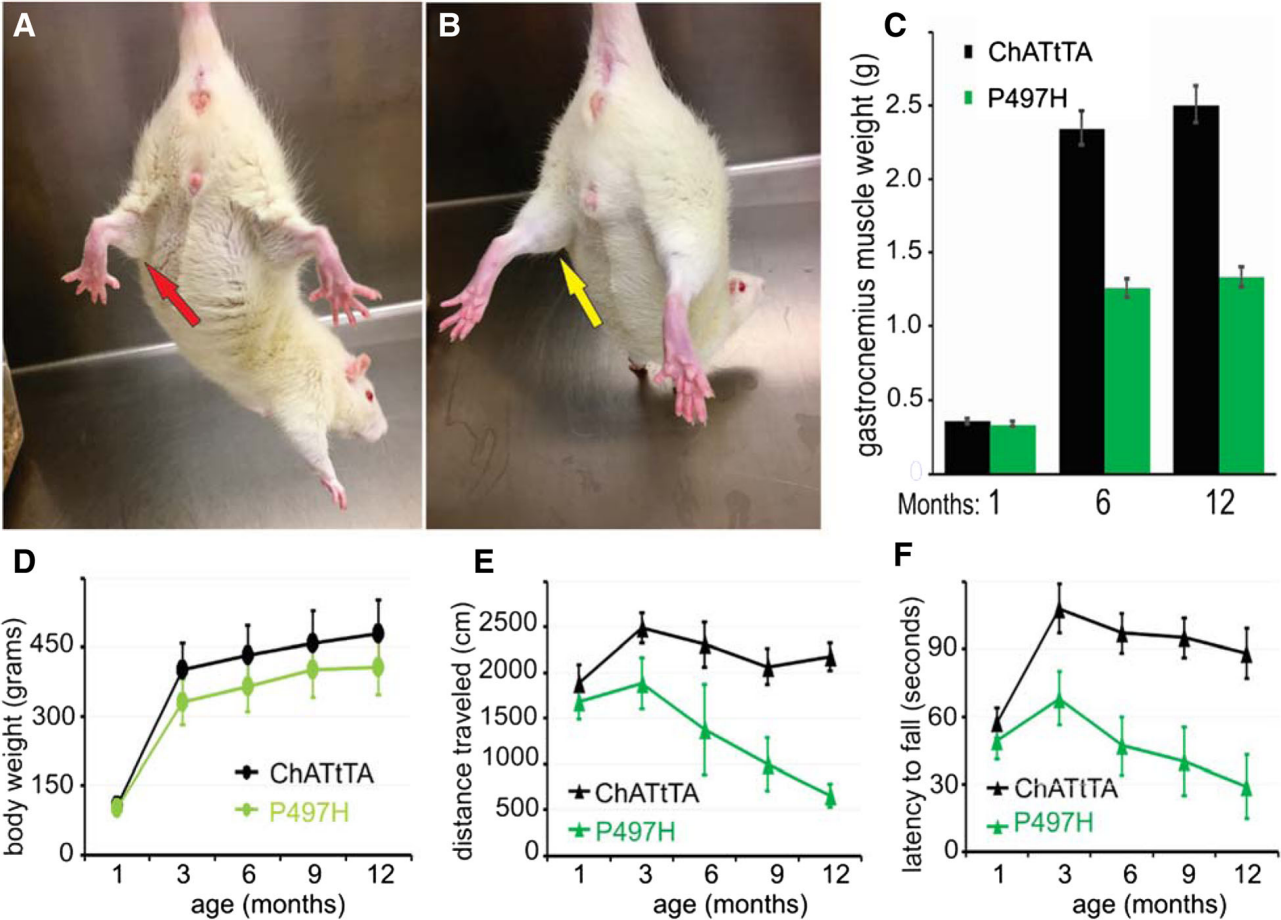
Overexpression of UBQLN2^P497H^ in motor neurons significantly decreased the mobility of rats. **a-b** Photos of dystrophic hind limbs in ChATtTA/UBQLN2^P497H^ (P497H) (**a**) but not in ChATtTA rats (**b**) at 12 months old. A red arrow points to the dystrophic hind limb in a P497H rat compared to a normal hind limb in a ChATtTA rat (yellow arrow). **c** A graph of the weights of gastrocnemius muscles in the P497H and ChATtTA male rats at 1 month, 6 months and 12 months old. **d** The body weights of P497H and ChATtTA male rats. **e** Analysis of the open-field test shows the distance traveled during a 10-min trial. **f** Analysis of the rotarod test shows the latency to fall. The data are reported as the mean ± standard deviation (*n* = 4, male rats were used)

Muscle weakness and decreased mobility are two key features of ALS [4, 28, 55]. As described in a TDP-43 rat model of ALS [16, 49], both muscular atrophy and loss of mobility occur as the disease progresses. Body weight was monitored and behavioral testing was conducted to investigate the role of mutant UBQLN2 in rats. As shown in Fig. 3, rats with mutant UBQLN2^P497H^ exhibited atrophic hind limbs (Fig. 3a) compared with ChATtTA rats (Fig. 3b), decreased body weight (Fig. 3d), reduced distance traveled in an open-field test (Fig. 3e), and reduced latency to fall from an accelerating rotarod device (Fig. 3f) compared to ChATtTA single transgenic rats. Severe muscle wasting (about 50%) in the gastrocnemius muscles was observed in mutant UBQLN2^P497H^ rats at 12 months old (Fig. 3c), whereas no significant alterations in either the tibialis or gastrocnemius muscles were observed at 1 month old (Additional file 1: Figure S2A-B). In addition, only mild weight loss (about 20%) was observed at 12 months old. Taken together, these findings indicate that overexpressing mutant UBQLN2^P497H^ in motor neurons cause the degeneration of both motor neurons and motor axons, progressive deficits in performance on both open-field and rotarod tests, and reductions in both body weight and gastrocnemius muscle weight.

**Fig. 4:**
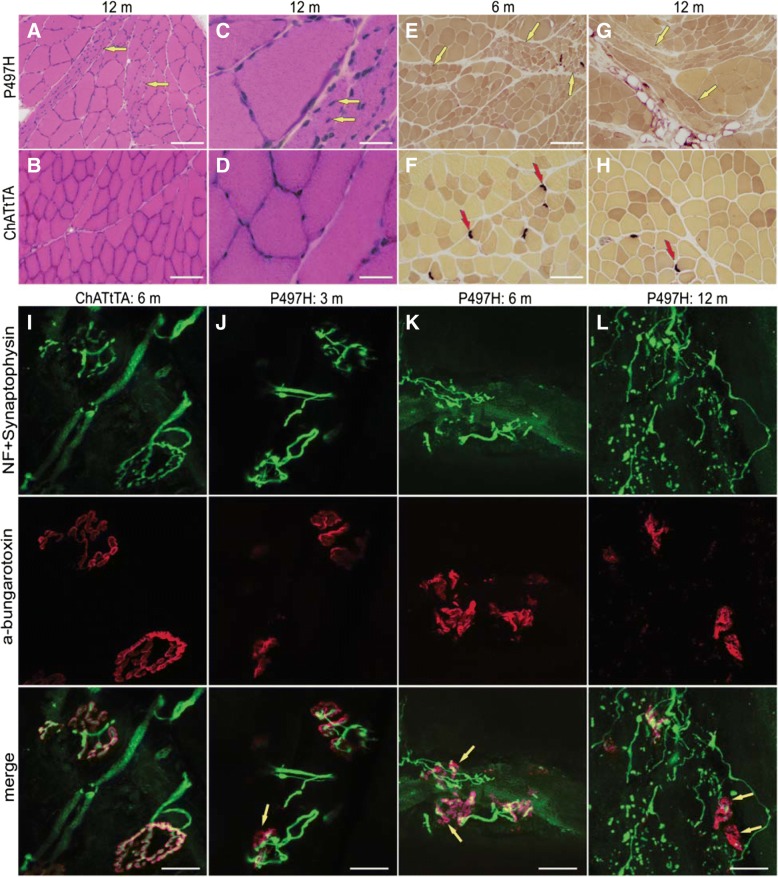
Denervation atrophy of skeletal muscles in ChATtTA/UBQLN2^P497H^ rats. **a-d** H&E staining shows groups of atrophic muscle fibers (**a,** arrows) and ectopic nuclei (**c,** arrows) in the gastrocnemius muscles of ChATtTA/UBQLN2^P497H^ (P497H) but not in ChATtTA single transgenic (ChATtTA) rats at 12 months old. **e-h** nonspecific esterase staining shows the grouped muscle atrophy (**e, g,** arrows) in the gastrocnemius muscles of P497H rats, and which are more severe atrophy in 12-month old rats than 6 months, but not in ChATtTA rats. The red arrows point to the neuromuscular junctions (NMJ). **i-l** The projection of confocal images reveals the innervation of NMJs in gastrocnemius muscles. The arrows (**j-l:** P497H) show that the motor end-plates are poorly innervated in P497H rats compared to ChATtTA rats (**I**), and the neurofilaments progressively form inclusions accompanied with the denervation of NMJ in P497H rats from 3 months to 12 months old. Scale bars: 100 μm (**a-b, e-h**), 30 μm (**c, d**), 20 μm (**i-l**)

We next examined whether mutant UBQLN2 affects muscle architecture via histology and immunostaining of the gastrocnemius muscles. H&E staining and histochemistry for nonspecific esterase revealed groups of muscle atrophy in the gastrocnemius muscles of ChATtTA/UBQLN2^P497H^ rats. No muscle atrophy was observed in ChATtTA single transgenic rats (Fig. 4a-h). H&E staining revealed that a substantial proportion of the nuclei were located internally in mutant UBQLN2^P497H^ rats compared to ChATtTA single transgenic rats. Both pH 4.6 and pH 10.4 ATPase staining also showed groups of atrophic muscle fibers, including type 1 and type 2 muscle fibers (Additional file 1: Figure S3, A and B). Immunofluorescence revealed that dystrophic myofibers were accumulated in the gastrocnemius muscles (Additional file 1: Figure S3, C-F), which further suggests that abnormal protein inclusion is a prominent feature of UBQLN2-related diseases [8, 10, 25].

Impairment of neuromuscular junctions is one of the earliest features to emerge in rodent models of ALS, and occurs at the presynaptic stage of the disease [33]. Longitudinal sections of gastrocnemius muscles were labeled with α-bungarotoxin (to label the motor end-plate) together with neurofilament and synaptophysin (to label the neuromuscular synapses). Confocal images showed a significant amount of denervated neuromuscular junctions (NMJ) in ChATtTA/UBQLN2^P497H^ rats as early as 3 months old but not in ChATtTA single transgenic rats (Fig. 4i-l), and a substantially increased number of the denervated NMJs with age (Additional file 1: Figure S2C), indicating that the neuromuscular synapses are more vulnerable than the motor neurons in rats that selectively express mutant UBQLN2^P497H^ in the spinal motor neurons.

Collectively, our results suggest that overexpression of mutant UBQLN2^P497H^ in the spinal motor neurons leads to motor neuron degeneration, impaired mobility, denervation atrophy of the skeletal muscles, and accumulation of myofibers in rats.

### Age-dependent reduction of autophagy-related proteins in UBQLN2^P497H^ rats

**Fig. 5:**
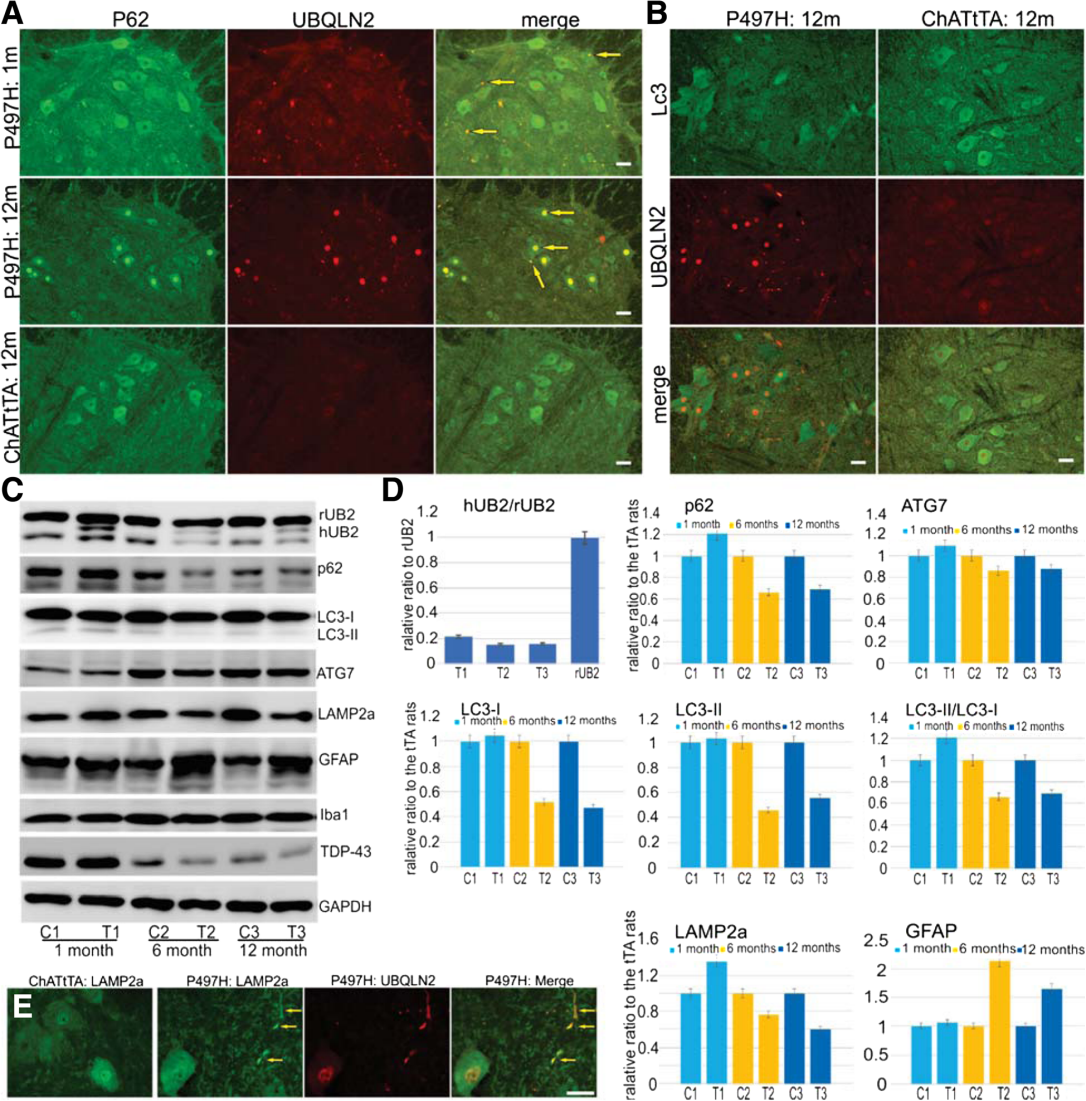
Accumulated UBQLN2 leads to the abnormal accumulation of autophagy-related proteins. **a** Double staining f p62 and UBQLN2 reveals that p62 progressively accumulates in the ventral horns of the spinal cord in ChATtTA/UBQLN2^P497H^ (P497H) but not in ChATtTA single transgenic (ChATtTA) rats. Accumulated p62 colocalizes with UBQLN2 inclusions. The arrows point to the protein inclusions. m: months. **b** Double staining of LC3 and UBQLN2 reveals weaker staining of LC3 in P497H rats than in ChATtTA rats. **c** Western blots of spinal cord lysates at 1 month, 6 months, and 12 months old probed with the indicated antibodies. **d** Graphs showing the quantification of human UBQLN2, P62, LAMP2a, LC3-I, LC3-II, LC3-II/LC3-I, GFAP, TDP-43 and Iba1, all of which are shown in (**c**). The data are reported as the mean ± standard deviation (*n* = 3, male rats were used). **e** Immunofluorescence staining shows that LAMP2a is accumulated in P497H but not ChATtTA rats at 6-month old. Scale bars: 50 μm

UBQLN2 plays a role in both the ubiquitin-proteasome and autophagy-lysosome systems [12, 24, 41]. To detect whether mutant UBQLN2^P497H^ affected the autophagy pathway, we examined two autophagy substrates, p62 and LC3, in the spinal cord of rats. Fluorescence staining revealed that the signals of diffused p62 and LC3 in the cytoplasm were weaker in the ventral horns in ChATtTA/UBQLN2^P497H^ rats compared to ChATtTA single transgenic rats (Fig. 5a, b). Furthermore, most cytoplasmic p62 was depleted at 12 months old (Fig. 5a). Concomitantly, p62 predominantly accumulated in the nucleus in ChATtTA/UBQLN2^P497H^ rats, which also colocalized with UBQLN2 inclusions (Fig. 5a) or ChAT inclusions (Additional file 1: Figure S4). We further confirmed these findings in the spinal cord lysates via immunoblotting (Fig. 5c, d). Interestingly, age-related reductions in LC3-I, LC3-II, and the ratio of LC3-II to LC3-I as well as in p62 were detected. Suppression of LC3-II and Beclin1 expression reflects impaired autophagy, and LC3-II levels are correlated with the extent of autophagosome formation [18]. In addition, in patients with AD, the reduction of functional p62 causes autophagy failure, which accelerates the development of AD [44, 51]. Our results are consistent with these findings and indicate that mutant UBQLN2 impairs autophagy in rats.

We also found reduced expression of another autophagy protein, ATG7, and a lysosomal protein, LAMP2a, which acts as a receptor in the lysosomal membrane for substrate proteins of chaperone-mediated autophagy [7]. LAMP2a was accumulated in the spinal cord sections of ChATtTA/UBQLN2^P497H^ rats and colocalized with UBQLN2 inclusions (Fig. 5e). Taken together, our findings suggest that mutant UBQLN2^P497H^ compromises the autophagy-lysosomal pathway in rats.

### TDP-43 pathology and ubiquitination in ChATtTA/UBQLN2^P497H^ rats

**Fig. 6:**
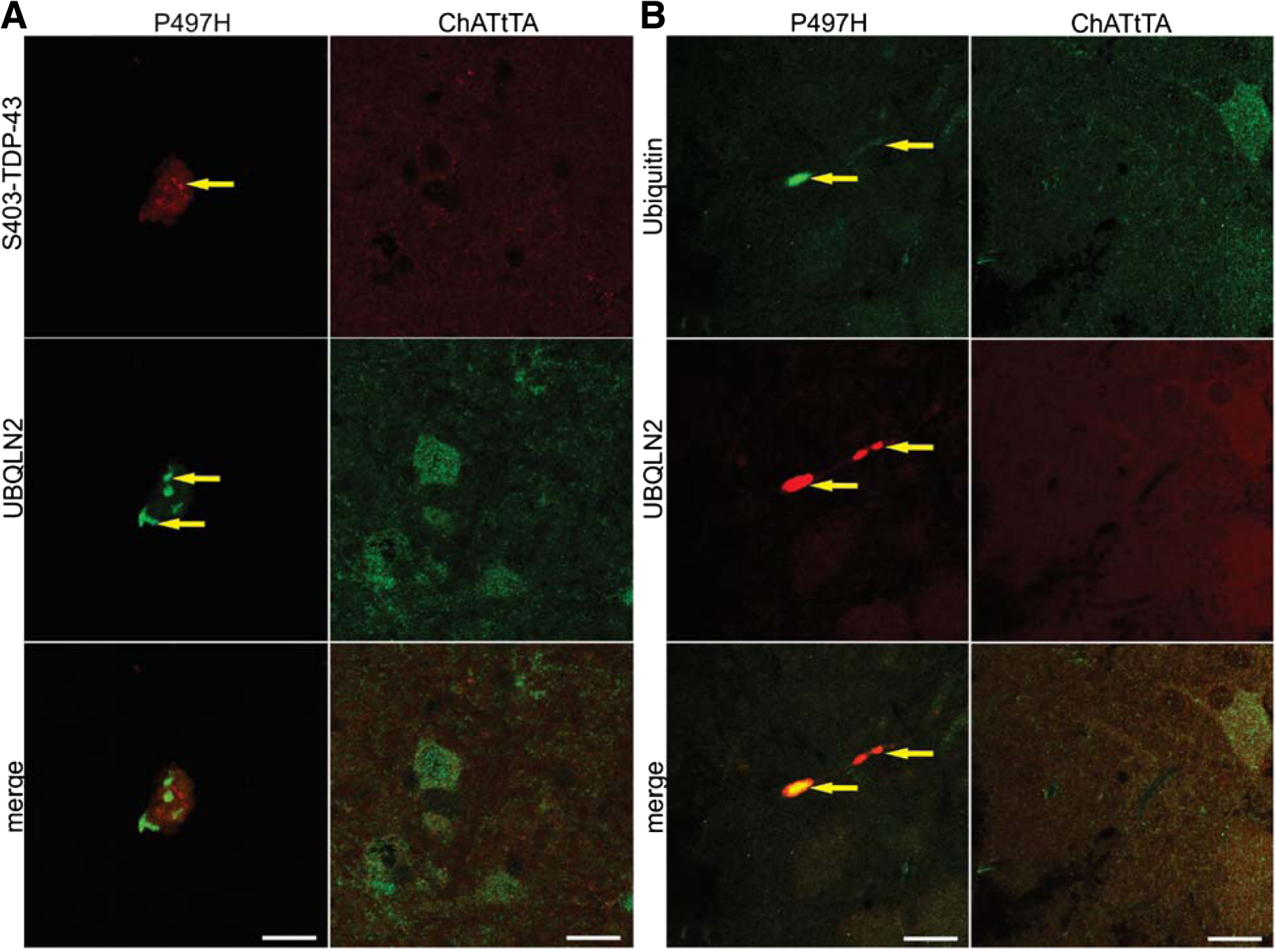
Accumulation of phosphorylated TDP-43 and ubiquitin in ChATtTA/UBQLN2^P497H^ rats. **a** Confocal images of phosphorlated TDP-43 (S403-TDP-43) and UBQLN2 staining in the spinal motor neurons of ChATtTA/UBQLN2^P497H^ rats shows that S403-TDP-43 positive inclusions do not colocalize with accumulated UBQLN2. **b** Confocal images of ubiquitin and UBQLN2 show that ubiquitin and UBQLN2 inclusions colocalize in P497H but not ChATtTA rats. The arrows point to the colocalized inclusions. Scale bars: 20 μm

Previous reports have shown that UBQLN2 inclusions co-exist with TDP-43 inclusions in patients with ALS [8, 41]. UBQLN2 also binds to the C-terminal region of TDP-43 [6]. Furthermore, pathologic TDP-43 is also hyper-phosphorylated [37]. We stained the coronal sections of spinal cord with phosphorylated TDP-43 antibody (S403-TDP-43), and found that phosphorylated TDP-43 was accumulated in ChATtTA/UBQLN2^P497H^ rats but did not colocalize with UBQLN2-positive inclusions (Fig. 6a). We did not detect any obvious reduction of nuclear TDP-43 by fluorescence staining (data not shown), but the total TDP-43 was decreased with age in ChATtTA/UBQLN2^P497H^ rats by immunoblot (Fig. 5c).

UBQLN2 binds to ubiquitin via its C-terminal ubiquitin associated domain [9, 21]. We therefore examined whether UBQLN2 inclusions promoted the accumulation of ubiquitin in ChATtTA/UBQLN2^P497H^ rats. Double immunofluorescence staining revealed that only minimal ubiquitin accumulation in the spinal cord of ChATtTA/UBQLN2^P497H^ rats occurred. In contrast, no accumulation was observed in ChATtTA rats (Fig. 6b). Accumulated ubiquitin colocalized with UBQLN2 inclusions, as shown by confocal single-scan images (Fig. 6b). Substantially increased GFAP expression was observed in the spinal cord lysates of ChATtTA/UBQLN2^P497H^ rats compared to ChATtTA rats (Fig. 5c, d), but there were no obvious alterations in microglia via immunoblotting with IBa1. Thus, our findings suggest that both the phosphorylation of TDP-43 and the ubiquitin inclusions that are observed in ALS patients could be replicated in our rat model.

### Mutant UBQLN2^P497H^ in astrocytes does not cause motor deficits in rats

**Fig. 7:**
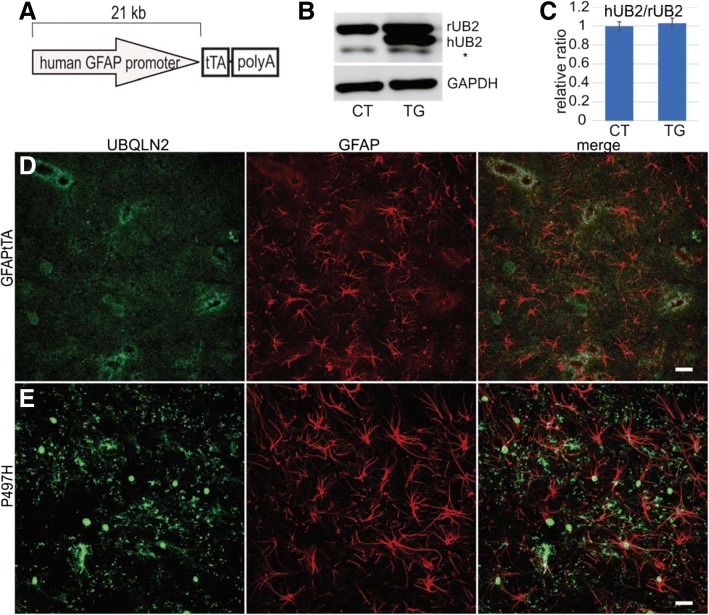
Expression of mutant UBQLN2^P497H^ in the astrocytes of rats. **a** A graph of the GFAPtTA construct. The trasgene is regulated by DOX. **b-c** Western blots show the relative expression level of human UBQLN2 in bigenic GFAPtTA/UBQLN2^P497H^ (P497H: TG) but not in control rats (GFAPtTA: CT) at 1 month old. rUB2: endogenous UBQLN2, hUB2: human UBQLN2, and the * indicates unknown bands. The data are reported as the mean ± standard deviation (*n* = 4, female rats were used). **d-e** The projection of confocal images shows the expression of human UBQLN2^P497H^ in the astrocytes of spinal cords in P497H but not in GFAPtTA rats. At 6 months old, a substantial proportion of UBQLN2 inclusions are mislocalized into the nuclei of astrocytes in P497H rats (**e**). Scale bars: 20 μm

To test whether mutant UBQLN2 expressed in astrocytes induces motor neuron death, we crossed TRE-UBQLN2^P497H^ rats with the GFAPtTA^#^2 line (Fig. 7a). As previously reported [49], the GFAPtTA^#^2 line is driven by a 21-kb human GFAP promoter, which induces restricted transgene expression in the astrocytes of spinal cord and brain. As in the ChATtTA/UBQLN2^P497H^ experiments, GFAPtTA/UBQLN2^P497H^ rats were deprived of DOX at birth. One month after DOX deprivation, immunoblotting revealed a substantial expression of human UBQLN2 in the spinal cord, which was similar in level to the expression of endogenous UBQLN2 (Fig. 7b, c). Compared to ChATtTA/UBQLN2^P497H^ rats, in which expression of human UBQLN2 accounted for about 20% of endogenous UBQLN2 expression, GFAPtTA/UBQLN2^P497H^ rats expressed higher amounts of transgene in the spinal cord. Immunofluorescence staining revealed accumulated UBQLN2 in the spinal cord astrocytes of GFAPtTA/UBQLN2^P497H^ but not in GFAPtTA rats at 6 months old (Fig. 7d-e).

**Fig. 8:**
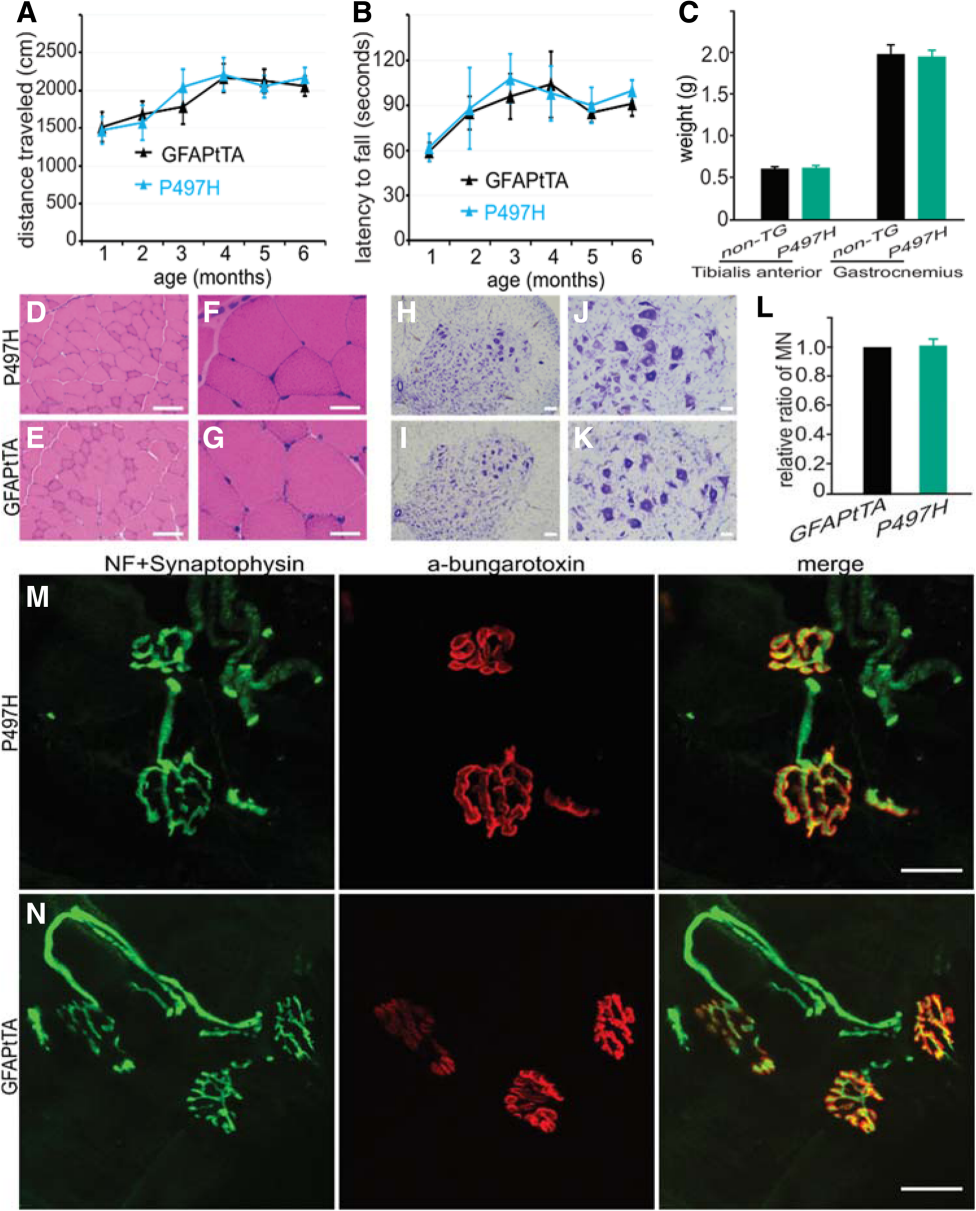
No motor phenotypes in GFAPtTA/UBQLN2^P497H^ rats. **a-b** Results of beavioral tests in GFAPtTA/UBQLN2^P497H^ bigenic (P497H) and GFAPtTA single transgenic rats. **c** The weights of tibialis anterior and gastrocnemius muscles at 6 months old in P497H and GFAPtTA female rats. The data are reported as the mean ± standard deviation (n = 4). **d-g** H&E staining shows the structures of gastrocnemius muscle in both P497H and GFAPtTA rats; **h-l** Cresyl violet staining shows the motor neurons in the ventral horn of the spinal cord. The quantification of motor neurons in the L3–5 spinal cord shows that there is no statistical difference between P497H and GFAPtTA rats (*n* = 4). **m-n** Confocal images show the structures of neuromuscular junctions (NMJ) in gastrocnemius muscles. The sections were stained with the presynaptic neuronal marker neurofilament (NF) and synaptophysin together with α-bungarotoxin to show the post-synaptic structures. Scale bars: 100 μm (**d**, **e**, **h**, **i**), 50 μm (**j**, **k**), 30 μm (**f**, **g**), 20 μm (**m**, **n**)

To examine their mobility, GFAPtTA/UBQLN2^P497H^ rats were subjected to monthly open-field and rotarod tests. No significant behavioral differences between GFAPtTA/UBQLN2^P497H^ rats and GFAPtTA rats were observed. In contrast to the rats that expressed mutant UBQLN2^P497H^ in motor neurons, no reduction in mobility, no motor neuron loss, no muscular atrophy, and no obvious denervation of neuromuscular junctions were observed in GFAPtTA/UBQLN2^P497H^ rats at 6 months old (Fig. 8). In addition to UBQLN2, however, both p62 and ubiquitin also were accumulated in the spinal cord astrocytes of GFAPtTA/UBQLN2^P497H^ but not GFAPtTA rats (Additional file 1: Figure S5). And we did not detect any pathological alterations in phosphorylated TDP-43, LC3, Lamp2a, and ATG7 (data not shown). These findings suggest that mutant UBQLN2 expression in astrocytes does not lead to motor phenotypes in 6-month old rats.

## Discussion

Mutations in UBQLN2 have been linked to ALS-FLTD, and the abnormal accumulation of UBQLN2 inclusions is a remarkable feature of pathological alterations linked to the UBQLN2 mutation [8, 46]. Several groups have recapitulated this specific pathological change in rodent models [10, 13, 25, 52]. No studies to date, however, have shown the effect of expressing mutant UBQLN2 either in the motor neurons or in the astrocytes to test whether mutant UBQLN2 expression leads to motor neuron degeneration in a cell-autonomous manner. To test this hypothesis, therefore, we created novel transgenic models expressing mutant UBQLN2^P497H^ in the spinal motor neurons or astrocytes in rats.

One recent report showed that transgenic mice expressing either the UBQLN2 P497S or P506T mutation developed both cognitive deficits and motor phenotypes, including progressive reduction of mobility, progressive loss of motor neurons in the spinal cord, and denervation of skeletal muscles as well as abnormal accumulation of UBQLN2 inclusions [25]. Similar motor phenotypes were observed in our novel ChATtTA/TRE-UBQLN2^P497H^ transgenic rats. In particular, denervation atrophy of the gastrocnemius muscles was observed as early as 3 months old, but loss of motor neurons was not detected at that age. The motor phenotypes, however, appeared for even low levels of mutant UBQLN2 (about 20% of the endogenous levels). In contrast, disease did not develop in SOD1^G93A^ mice until the levels of mutant SOD1 were three times that of endogenous SOD1 protein [11]. Moreover, both Gorrie et al. [10] and Hjerpe et al. [13] reported progressive accumulation of UBQLN2 inclusions and progressive cognitive deficits in mice expressing either the UBQLN2 P497H or P506T mutation. All these findings indicate that mutant UBQLN2 leads to neuron degeneration in rodent models. Furthermore, the abnormal accumulation of UBQLN2 inclusions is a remarkable pathological feature of UBQLN2-related diseases.

Similar findings have been reported in transgenic rats expressing mutant TDP43 (M337 V substitution) in the spinal motor neurons, which causes rapid degeneration of motor neurons and paralysis [16]. In contrast, transgenic mice expressing mutant SOD1 in the motor neurons do not develop motor phenotypes [29, 50]. The causes of these differences remain unknown. One possible reason for these differences is that different disease mechanisms may underlie UBQLN2, TDP-43, and SOD1 genes. For example, UBQLN2 involves protein degradation via both autophagy and the ubiquitin-proteasome pathway [9, 13, 36, 41, 53]. The overexpression of UBQLN2^P497H^ in the spinal motor neurons caused the autophagy substrate p62 to progressively accumulate as well as colocalize with both UBQLN2 and ChAT inclusions in the ventral horn of spinal cord in ChATtTA/UBQLN2^P497H^ rats, which is similar to the results from transgenic rats expressing UBQLN2 in forebrain neurons [14, 52]. At 12 months old, p62 accumulated predominantly in the nucleus and cytoplasmic p62 was mostly depleted. Under physiological conditions, however, p62 is commonly considered a cytoplasmic protein. p62 contains two nuclear localization signals (NLS) and a nuclear export signal, however, and it has been confirmed that p62 also shuttles between the nucleus and cytoplasm, a process that is regulated by the phosphorylation of NLS [38]. The mislocalization of p62, as observed in our study, may be the underlying cause of the abnormal functions observed in our ChATtTA/UBQLN2^P497H^ rats. Total p62 was increased in ChATtTA/UBQLN2^P497H^ rats at 1 month old, which is similar to the findings in rats expressing mutant UBQLN2 in the forebrain [52]. As an autophagy substrate, p62 is essential to neurons [22], and mutations in p62 have been linked to ALS and FTLD [42]. In mouse models, loss of p62 leads to neurodegeneration [40]. Our finding that accumulated p62 colocalizes with UBQLN2 inclusions is similar to our previous reports in other rat models [14, 52]. These findings imply that the two disease genes may share similar mechanisms underlying neurodegeneration. Specifically, mutant UBQLN2^P497H^ may compromise the functions of autophagy, leading to abnormal protein accumulations of UBQLN2, p62, and others.

Although p62 has been used as one indicator of autophagy [3, 30, 43], autophagic flux should be measured by an LC3 turnover assay in addition to p62. LC3-II is one isoform of LC3, and is widely used to measure the autophagic process [22, 47]. The suppression of LC3-II expression reflects impaired autophagy, and the amount of LC3-II is correlated with the extent of autophagosome formation [18]. ATG7 is another autophagy component that is essential for autophagosome formation. The loss of ATG7 leads to the reduction of autophagy in mice [23]. In our ChATtTA/UBQLN2^P497H^ rats, both LC3 and ATG7 were accumulated at 1 month old but decreased substantially after 6-month old. Similarly, the lysosomal membrane protein LAMP2a also was accumulated and colocalized with UBQLN2 inclusions in 12-month old ChATtTA/UBQLN2^P497H^ rats. Consistent with these findings, the selective loss of LAMP2A protein directly correlated with increased levels of α-synuclein in early Parkinson’s disease [34]. All these results suggest that mutant UBQLN2^P497H^ compromises autophagy-lysosomal pathways in an age-dependent manner. Moreover, the decrease of several core ATG proteins suggests that mutant UBQLN2^P497H^ is more likely to suppress autophagy at upstream stages.

The hyperphosphorylated form of TDP-43 has been identified as a core component of cytosolic inclusions in sporadic ALS [1, 37]. In ChATtTA/UBQLN2^P497H^ rats, relatively little phosphorylated TDP-43 was detected in the spinal cord and did not colocalize with UBQLN2 inclusions. In contrast, accumulated ubiquitin was colocalized with UBQLN2 inclusions. Ubiquitin accumulation is one of the key pathological alterations in neurodegenerative diseases, and ubiquitin accumulation may correlate with neurodegeneration in our rats. In contrast, the expression of the astrocyte marker GFAP was elevated in our rats, but no significant changes in IBa1, a marker of microglia, were observed. These findings are different from those of other rodent models, in which increased expression in both astrocytes and microglia have been observed [16, 17, 25, 52]. This difference may be due to the slow progression of the disease and a mild loss of motor neurons in ChATtTA/UBQLN2^P497H^ rats compared to other rat models, indicating that astrocytes are more sensitive to stress conditions than microglia in our mutant UBQLN2^P497H^ rats.

We did not observe any motor deficits at 6-month old GFAPtTA/UBQLN2^P497H^ rats, which is not consistent with the motor phenotypes observed in mutant TDP-43 or mutant SOD1 rodent models [26, 35, 49]. Mutant UBQLN2 protein expression was higher in the spinal cord of GFAPtTA/UBQLN2^P497H^ rats compared to ChATtTA/UBQLN2^P497H^ rats. These findings suggest that phenotypes caused by mutant UBQLN2^P497H^ are not dependent solely on the expression level of mutant proteins in motor neurons and astrocytes in rats. It would be important to investigate whether any disease phenotypes can be induced in older rats (up to 18 months old) expressing mutant UBQLN2^P497H^ in astrocytes. In addition, future studies are needed to examine whether mutant UBQLN2 will initiate motor neuron degeneration in a non-cell autonomous manner when overexpressing mutant UBQLN2 in other non-neuronal cells, such as microglia or oligodendrocytes.

## Conclusions

Our results showed that mutant UBQLN2^P497H^ selectively expressed in motor neurons other than astrocytes leads to several key features of motor neuron disease in rats, including abnormal accumulation of UBQLN2, p62, and ChAT; mobility impairment; motor neuron degeneration; and reductions in several core autophagy-related proteins. This study indicates that expressing mutant UBQLN2 in motor neurons leads to progressive motor deficits and impairment of the autophagy-lysosomal pathway, but that overexpression of mutant UBQLN2 in astrocytes alone is not sufficient to develop motor phenotypes or defective autophagy.

## Additional file

Additional file 1: **Figure S1.** Similar expression of UBQLN2 in male and female non-transgenic rats. Western blotting showed the endogenous UBQLN2 (rUB2) had no differential expression between male and female non-transgenic rats at the age of 90 days. The upper graph showed the relative ratios of endogenous UBQLN2 between female and male among different tissues. (M: male, F: female. “*” denotes non-specific band). Data are shown as mean ± s.d. (*n* = 3). **Figure S2.** Muscle structures in rats. (**A-B**), H&E staining showed no alteration was observed in both tibialis anterior and gastrocnemius muscles of ChATtTA/UBQLN2P497H rats (P497H) compared with ChATtTA single transgenic rats (ChATtTA) at 1 month old. Panel A: 4x objective, and Panel B: 10x objective. Scale bars: A (250 μm), B (100 μm). (**C**), Quantification of the impaired neuromuscular junctions in P497H rats at indicated ages, which are the same rats shown in Fig. 4i-l. (> 20 NMJs were counted randomly for each rats). **Figure S3.** Accumulation of myofibers in ChATtTA/UBQLN2P497H rats. (**A-B**), Both pH 4.6 and pH 10.4 ATPase staining revealed groups of atrophic muscle fibers in gastrocnemius muscles of ChATtTA/UBQLN2P497H rats (P497H) at 12 months old, not in the age-matched ChATtTA single transgenic rats (ChATtTA). (**C-F**), Immunofluorescent staining of myofibers (MYH-S and MYH-1) and DMD (a plasma membrane protein) showed the atrophic myofibers accumulated in gastrocnemius muscles of P497H rats, not in ChATtTA rats. Arrows point to groups of myofiber atrophy. Scale bars: 100 μm. **Figure S4.** The colocalization of the accumulated ChAT and p62 in rats. (**A-C**), Double staining of p62 and ChAT revealed the accumulation of p62 in ChATtTA/UBQLN2P497H rats (P497H, arrows point to the accumulations), not in ChATtTA single transgenic rats (ChATtTA). At 12 months old, a substantial proportion of ChAT mislocalilzed into nuclei and also colocalized with the p62 inclusions (**C**). Scale bars: 100 μm. **Figure S5.** Accumulations of P62 and ubiquitin in GFAPtTA/UBQLN2P497H rats. (**A**), Double staining of P62 and GFAP revealed the accumulations of P62 were colocalized with astrocytes in GFAPtTA/UBQLN2P497H rats (P497H, arrows point to the colocalizations of inclusions), not in GFAPtTA single transgenic rats (GFAPtTA). (**B**), The projected confocal images of ubiquitin and GFAP showed the colocalization of the accumulated ubiquitin and astrocytes in P497H rats (arrows point to the colocalizations), not in GFAPtTA rats. Scale bars: 30 μm (**A**), 20 μm (**B**). (PDF 1240 kb)

## Abbreviations

AD: Alzheimer’s disease
ALS: Amyotrophic lateral sclerosis
C9ORF72: Chromosome 9 open reading frame 72
CHAT: Choline acetyltransferase
CNS: Central nervous system
dox: Doxycycline
FTLD: Frontotemporal lobar degeneration
UBA: Ubiquitin associated domain
UBL: Ubiquitin-like domain
UBQLN2: Ubiquilin2
