# Supplementary material for: Mutant UBQLN2P497H in motor neurons leads to ALS-like phenotypes and defective autophagy in rats
Source: Acta Neuropathol Commun. 2018 Nov 8;6:122. doi: 10.1186/s40478-018-0627-9 (PMC6225656; doi:10.1186/s40478-018-0627-9)
Supplement: Supplementary file 1 — Figure S1. Similar expression of UBQLN2 in male and female non-transgenic rats. Western blotting showed the endogenous UBQLN2 (rUB2) had no differential expression between male and female non-transgenic rats at the age of 90 days. The upper graph showed the relative ratios of endogenous UBQLN2 between female and male among different tissues. (M: male, F: female. “*” denotes non-specific band). Data are shown as mean ± s.d. (n = 3). Figure S2. Muscle structures in rats. (A-B), H&E staining showed no alteration was observed in both tibialis anterior and gastrocnemius muscles of ChATtTA/UBQLN2P497H rats (P497H) compared with ChATtTA single transgenic rats (ChATtTA) at 1 month old. Panel A: 4x objective, and Panel B: 10x objective. Scale bars: A (250 μm), B (100 μm). (C), Quantification of the impaired neuromuscular junctions in P497H rats at indicated ages, which are the same rats shown in Fig. 4i-l. (> 20 NMJs were counted randomly for each rats). Figure S3. Accumulation of myofibers in ChATtTA/UBQLN2P497H rats. (A-B), Both pH 4.6 and pH 10.4 ATPase staining revealed groups of atrophic muscle fibers in gastrocnemius muscles of ChATtTA/UBQLN2P497H rats (P497H) at 12 months old, not in the age-matched ChATtTA single transgenic rats (ChATtTA). (C-F), Immunofluorescent staining of myofibers (MYH-S and MYH-1) and DMD (a plasma membrane protein) showed the atrophic myofibers accumulated in gastrocnemius muscles of P497H rats, not in ChATtTA rats. Arrows point to groups of myofiber atrophy. Scale bars: 100 μm. Figure S4. The colocalization of the accumulated ChAT and p62 in rats. (A-C), Double staining of p62 and ChAT revealed the accumulation of p62 in ChATtTA/UBQLN2P497H rats (P497H, arrows point to the accumulations), not in ChATtTA single transgenic rats (ChATtTA). At 12 months old, a substantial proportion of ChAT mislocalilzed into nuclei and also colocalized with the p62 inclusions (C). Scale bars: 100 μm. Figure S5. Accumulations of P62 and ubiquitin in GFAPtTA/UBQLN [file 40478_2018_627_MOESM1_ESM.pdf]

Mutant UBQLN2^{P497H} in motor neurons leads to ALS-like phenotypes and defective autophagy in rats

(Supplimentary Figures)

Tianhong Chen[1, 5], Bo Huang[2, 3, 5], Xinglong Shi[1], Limo Gao[4], Cao Huang[1]

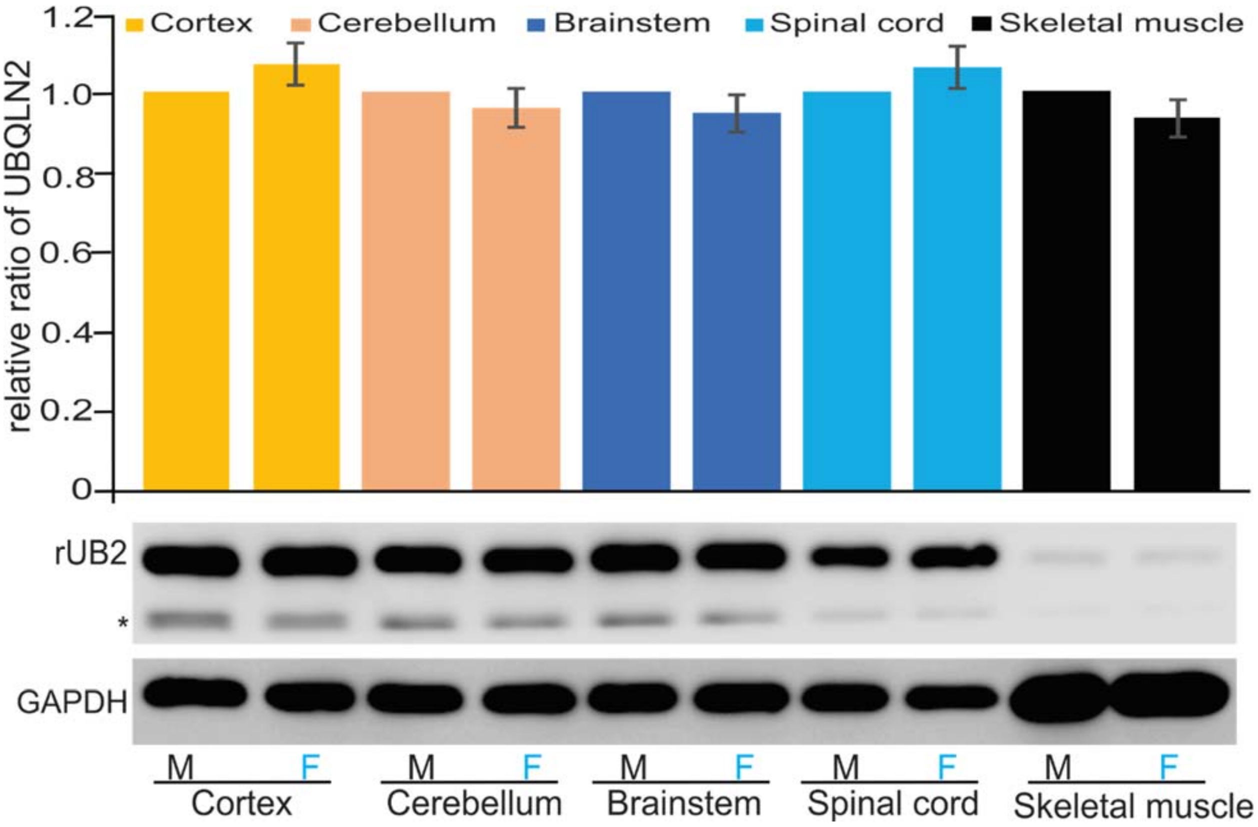

Figure S1: Similar expression of UBQLN2 in male and female non-transgenic rats. Western blotting showed the endogenous UBQLN2 (rUB2) had no differential expression between male and female non-transgenic rats at the age of 90 days. The upper graph showed the relative ratios of endogenous UBQLN2 between female and male among different tissues. (M: male, F: female. "*" denotes non-specific band). Data are shown as mean ± s.d. (n=3)

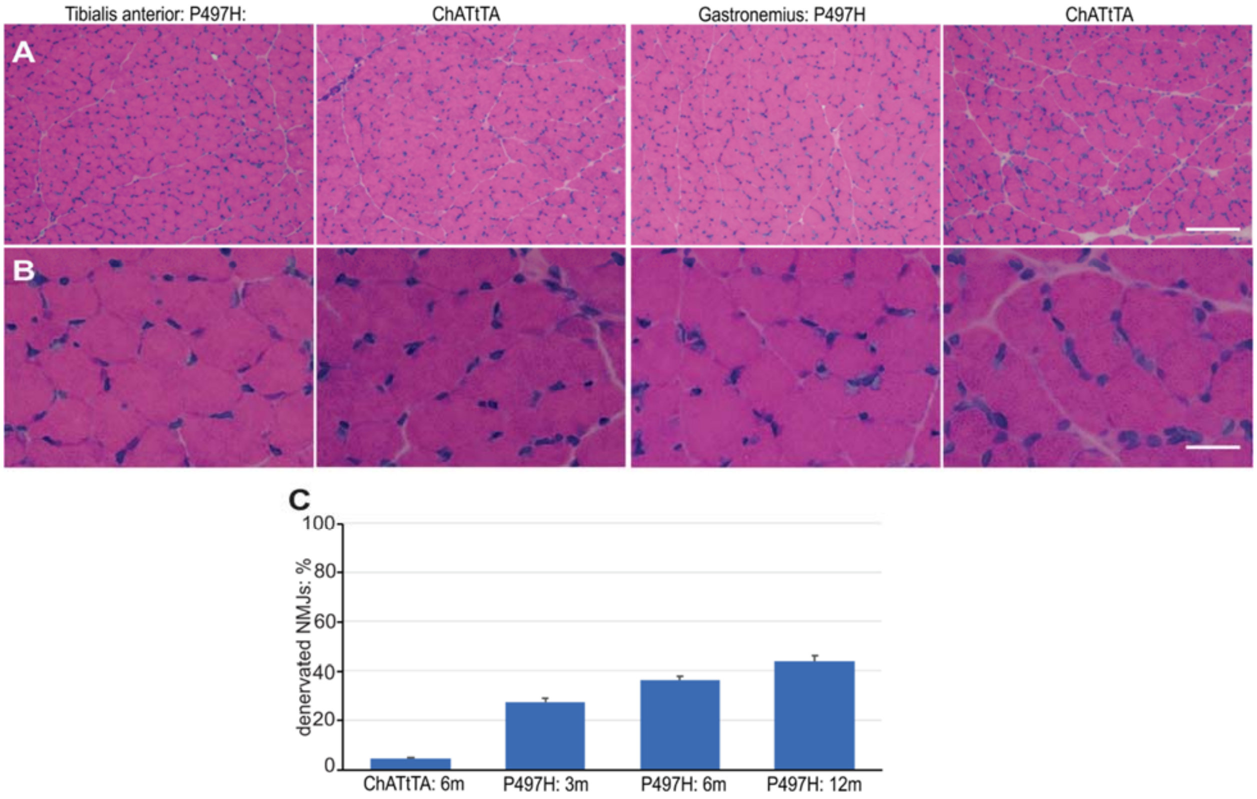

Figure S2: Muscle structures in rats.

(**A-B**), H&E staining showed no alteration was observed in both tibialis anterior and gastrocnemius muscles of ChATtTA/UBQLN2^{P497H} rats (P497H) compared with ChATtTA single transgenic rats (ChATtTA) at 1 month old. Panel A: 4x objective, and Panel B: 10x objective. Scale bars: A (250 µm), B (100 µm). (**C**), Quantification of the impaired neuromuscular junctions in P497H rats at indicated ages, which are the same rats shown in Figure 4I-L. (>20 NMJs were counted randomly for each rats)

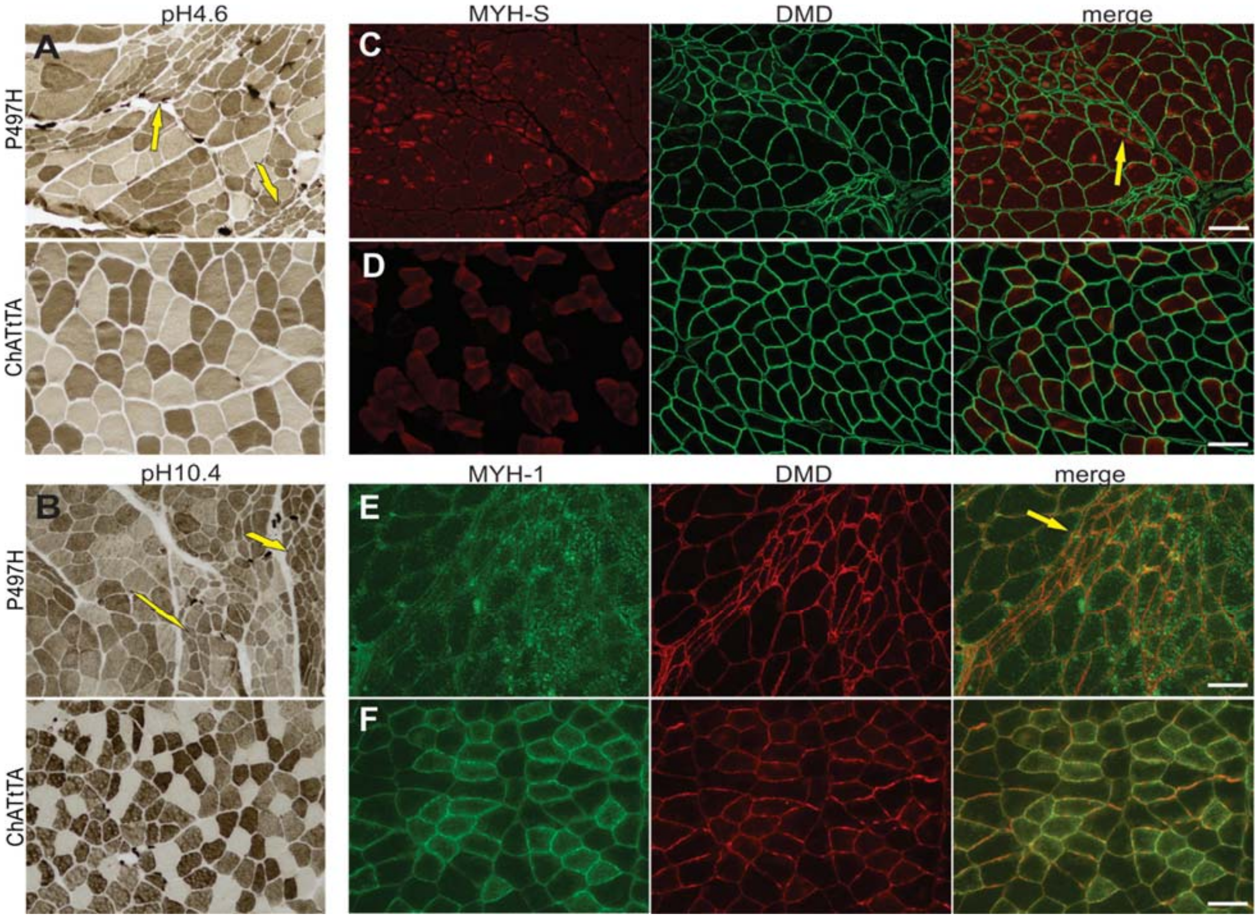

Figure S3: Accumulation of myofibers in ChATtTA/UBQLN2^{P497H} rats.

(**A-B**), Both pH4.6 and pH10.4 ATPase staining revealed groups of atrophic muscle fibers in gastrocnemius muscles of ChATtTA/UBQLN2^{P497H} rats (P497H) at 12 months old, not in the age-matched ChATtTA single transgenic rats (ChATtTA). (**C-F**), Immunofluorescent staining of myofibers (MYH-S and MYH-1) and DMD (a plasma membrane protein) showed the atrophic myofibers accumulated in gastrocnemius muscles of P497H rats, not in ChATtTA rats. Arrows point to groups of myofiber atrophy. Scale bars: 100 µm.

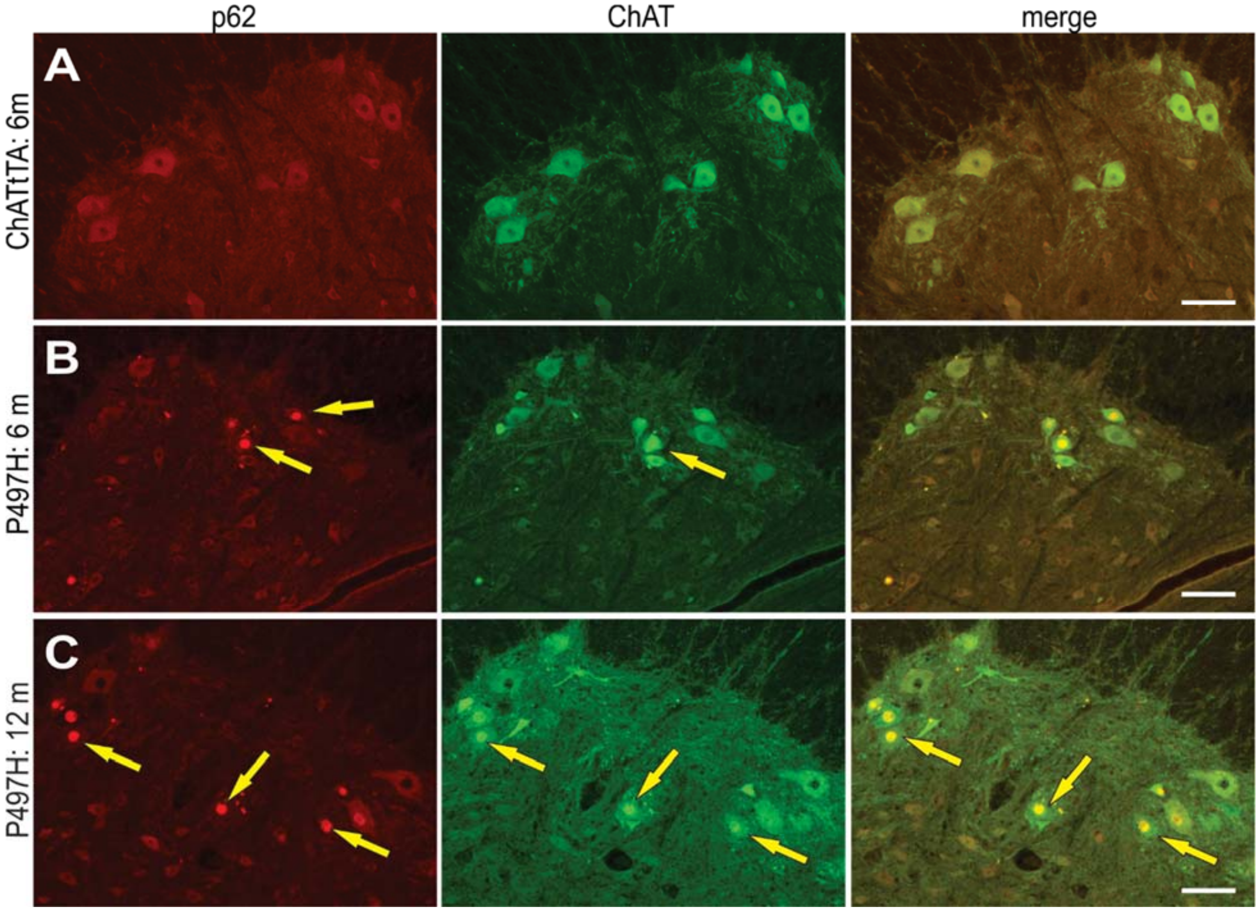

Figure S4: The colocalization of the accumulated ChAT and p62 in rats.

(**A-C**), Double staining of p62 and ChAT revealed the accumulation of p62 in ChATtTA/UBQLN2^{P497H} rats (P497H, arrows point to the accumulations), not in ChATtTA single transgenic rats (ChATtTA). At 12 months old, a substantial proportion of ChAT mislocalilzed into nuclei and also colocalized with the p62 inclusions (**C**). Scale bars: 100 µm.

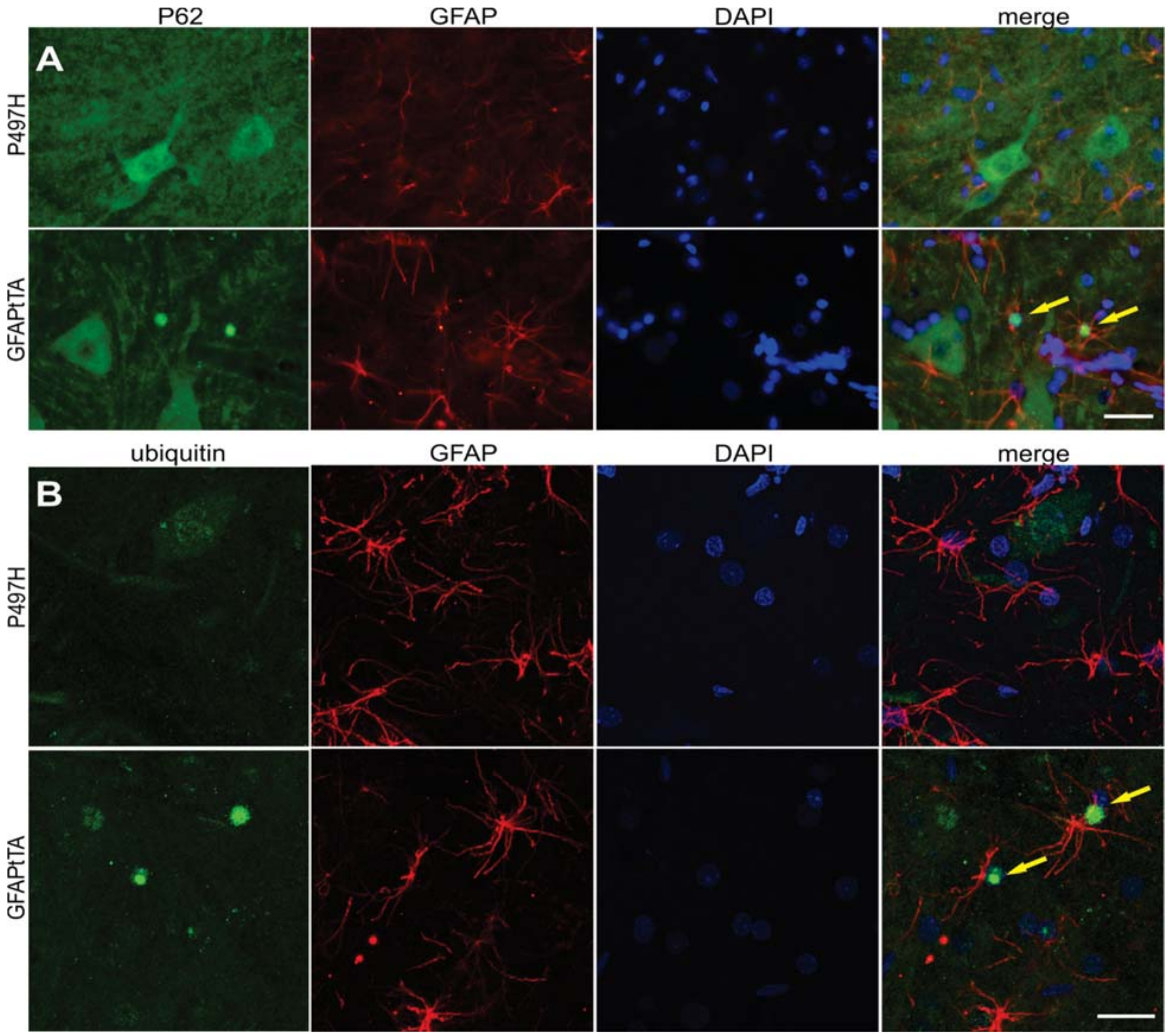

Figure S5: Accumulations of P62 and ubiquitin in GFAPtTA/UBQLN2^{P497H} rats.

(**A**), Double staining of P62 and GFAP revealed the accumulations of P62 were colocalized with astrocytes in GFAPtTA/UBQLN2^{P497H} rats (P497H, arrows point to the colocalizations of inclusions), not in GFAPtTA single transgenic rats (GFAPtTA). (**B**), The projected confocal images of ubiquitin and GFAP showed the colocalization of the accumulated ubiquitin and astrocytes in P497H rats (arrows point to the colocalizations), not in GFAPtTA rats. Scale bars: 30 µm (**A**), 20 µm (**B**).
